# Supplementary material for: Structural annotation of unknown molecules in a miniaturized mass spectrometer based on a transformer enabled fragment tree method
Source: Commun Chem. 2024 May 13;7:109. doi: 10.1038/s42004-024-01189-0 (PMC11091078; doi:10.1038/s42004-024-01189-0)
Supplement: Supplementary file 2 — Supplementary Information [file 42004_2024_1189_MOESM2_ESM.pdf]

Supplementary Information

Structural annotation of unknown molecules in a miniaturized mass spectrometer based on a transformer enabled fragment tree method

Yiming Yang[1], Shuang Sun[1], Shuyuan Yang[1], Qin Yang[1], Xinqiong Lu[2], Xiaohao Wang[1], Quan Yu[1], Xinming Huo[3,*], Xiang Qian[1,*]

1. Shenzhen International Graduate School, Tsinghua University, Shenzhen, 518055, China
2. Shenzhen Chin Instrument Co. Ltd., Shenzhen, 518055, China
3. School of Biomedical Engineering, Shenzhen Campus of Sun Yat-sen University, Shenzhen 518107, China

* Corresponding Author: qian.xiang@sz.tsinghua.edu.cn, huoxm@mail.sysu.edu.cn

Supplementary Table S1: Dissociation and rearrangement rules used in the Recap method

Flavanols:<br>"[#6:1]@[#6:2](=[O:3])@[#6:4]>>[#6:1]-[#6:4].[#6H0:2](=[O:3])",<br>"[c:1]-[C:2](=[C:3]-[O:4])-[c:5]:[c:6]:[c:7]:[c:8](-[O:9])@[c:10]@[c:11]>>[c:1]-[C:2]=[C]1[C]=[C]-[C](=[O])-[C:10]=[C:11]1.[C]-[O]",<br>"[c:1]@[Cr4:2]@[Cr4:3]@[c:4]>>[C:2]-[C:3].[c:1]-[c:4]",<br>"[Cr4:1]-[c:2]>>[Cr4:1].[c:2]",
Stilbene:<br>"[c:1]-[C:2]=[C:3]-[c:4]>>[C]=[C]-[C:2]=[C:3]-[c:4]",<br>"[cr6:1]@[cr6:2](-[O:7])@[cr6:3]@[cr6:4]@[cr6:5]@[cr6:6]>>[C:1]-[C:3]-[C:4]=[C:5]-[C:6]",<br>"[C:1]1=[C:3]-[C:4]-[C:5]=[C:6]1>>[C:1]1-[C:3]=[C:6]1",
Commonly rules:<br>"[#7;+0;D2,D3:1]-!@[S:2](=[O:3])=[O:4]>>[#7:1].[S:2](=[O:3])=[O:4]",<br>"[C:1]=!@[C:2]>>[C+:1].[C+:2]",<br>"[#6H2:1]-!@[#6H1:2]-[O;-1]>>[#6H1:1]=,:[#6+:2]",<br>"[#6H2:1]-!@[#6H2:2]-[O;-1]>>[#6H1:1]=,:[#6H1+:2]",<br>"[#6H2:1]-!@[#6H1:2]-[O]>>[#6H1:1]=,:[#6H1:2]",<br>"[#6H2:1]-!@[#6H2:2]-[O]>>[#6H1:1]=,:[#6H2:2]",<br>"[#6H1:1]-!@[#6H1:2]-[O]>>[#6H0:1]=,:[#6H1:2]",<br>"[#6H1:1]-!@[#6H1:2]-[O]>>[#6H0:1]=,:[#6H1:2]",<br>"[C;+0:1]-!@[O;+0,+0:2]>>[C;+0:1].[O;+0,+0:2]",<br>"[c:1]-[c:2](=O)-[c:3]>>[c:1]-[c:3]",<br>"[CH;+0:1]-[OH:2]>>[CH0;+1:1].[O;-1:2]",<br>"[#6;+0:1]-!@[N;+0,+0:2]>>[#6+1:1].[N;+1:2]",<br>"[C;0:1]-!@[O;-1,+0:2]>>[C;+1:1].[O;-1:2]",<br>"[C;0:1]-!@[O;-1,+0:2]>>[C;+1:1].[O;0:2]",<br>"[C;+1:1]-!@[N;+0,+0:2]>>[C;+2:1].[N;+1:2]",<br>"[C;+1:1]-!@[C;+1,+0:2]>>[C;+1:1].[C;+1:2]",<br>"[C;+0:1]-!@[N;+1,+0:2]>>[C;+1:1].[N;+2:2]",<br>"[C;+0:1]-!@[C;+0:2]>>[C;+1:1].[C;+1:2]",<br>"[C;+0:1]-!@[C;+0:2]>>[C;+0:1].[C;+0:2]",<br>"[C;+0:1]-!@[C;+1:2]>>[C;+1:1].[C;+2:2]",<br>"[C;+0:1]-!@[S;+0:2]>>[C;+1:1].[S;+1:2]",<br>"[c;+0:1]-[O;+0:2]>>[c;+0:1].[O;-1:2]",<br>"[c;+0:1]-[C;+0:2]>>[c;+0:1].[C;+0:2]",

Supplementary Note 1：Algorithm for Enhancing Mass Spectrometer Resolution

Due to the instability of the equipment hardware and the variability of the operating environment, the system error of the on-site mass spectrometer has significant instability and nonlinearity. This experiment eliminates random errors by averaging multiple spectra to calibrate the left and right drift of the mass spectrum and performs overall calibration of the peak shape through deconvolution.

In Supplementary Equation 1, the mass spectrometry measurement, denoted as *y*, can be described as the result of a convolution operation between the theoretical isotope distribution, y_0, and the actual peak function, *p*.

$$y = y_0 \otimes p \tag{1}$$

The peak function, *p*, can be mathematically transformed into a well-defined function, denoted as *d*, for instance, through convolution with the filter function, *f*. This filter function serves the purpose of calibrating m/z displacement and rectifying kurtosis distortion, as delineated in Supplementary Equation 2.

$$d = p \otimes f \tag{2}$$

By organizing these two equations, we can derive Supplementary Equation 3:

$$y_0 \otimes d = y \otimes f \tag{3}$$

By Supplementary Equation 3, given the known theoretical isotope distribution, theoretical peak shape, and the actual mass spectrometry signal, the calibration filter, denoted as f, can be computed through deconvolution. In the realm of mass spectrometers, Gaussian or normal error distributions have been established as reliable approximations for describing the mass function. Consequently, we opt for the Gaussian function as the theoretical peak shape function. This filter is subsequently employed for the calibration of the initial mass spectrometry signal, y, resulting in the calibration mass spectrometry signal, r, as depicted in Supplementary Equation 4.

$$y \otimes f = r \tag{4}$$

The procedure for computing a calibration filter involves several steps. Initially, a spectrum of a well-defined substance is acquired within the mass spectrometry. Subsequently, according to its chemical formula, the theoretical isotope distribution of this substance is calculated. Finally, deconvolution operations are carried out by employing the Richardson-Lucy algorithm on the actual spectrum of the substance and the theoretical isotope distribution to derive the calibration filter. We employ the Richardson-Lucy algorithm to compute the calibration filter, assuming that the noise conforms to a Poisson distribution through a maximum likelihood approach. The iterative principle involves presuming that the sample's probability density function adheres to a Gaussian distribution. Leveraging Bayesian probability, Supplementary Equation 5 expresses the probability of theoretical mass spectrum occurring while estimating the filter as f.

$$P\big((y_0 * d)|f)\big) = \prod\big(\frac{(f * y)^{y_0 * d} e^{-f * y}}{y_0 * d}\big) \tag{5}$$

The maximum value of $P\big((y_0 * d)|f)\big)$ in Supplementary Equation 5 can be determined by minimizing $-lnP\big((y_0 * d)|f)\big)$, as shown in Supplementary Equation 6.

$$-lnP\big((y_0 * d)|f)\big) = \sum(-(y_0 * d)\ln(f * y) + f * y) \tag{6}$$

Since $-lnP\big((y_0 * d)|f)\big)$ is a convex function, the minimum value is equivalent to the corresponding value when its derivative is 0, as shown in Supplementary equation 7, where $y(-i)$ is the flip of $y(i)$.

$$\frac{y_0 * d}{(f^k * y)} * y(-i) = 1 \tag{7}$$

Based on this, the iterative equation for f can be derived, as presented in Supplementary Equation 8. As the number of iterations k decreases noise may become amplified. In our experimental work, we have selected an iteration number of 20.

$$f^{k+1} = \left\{\frac{y_0 * d}{(f^k * y)} * y(-i)\right\} f^k \tag{8}$$

The calibration filter is applied through convolution to the actual mass spectrum of the substance being measured. This process yields a calibrated spectrum with enhanced resolution.

When calculating the theoretical isotope distribution of substances, according to Rockwood's convolution algorithm

based on isotope ratios, the isotope distribution can be calculated by gradually adding the isotope ratio information of a specific atom to the entire molecule. Alternatively, we can represent the isotopic function of atoms as δ functions, associating them with convolution, which entails gradually convolving the isotope function of a specific atom into the entire molecule. According to the time-domain convolution theorem based on the Fourier transform, the transformation from time-domain convolution to frequency-domain convolution is equivalent to multiplication. Therefore, the isotope distribution in the mass domain can be converted to the μ domain for processing and subsequently transformed back to the mass domain.

Construct the μ domain function for each element, predicated on its atomic mass and corresponding abundance. As an illustration, consider the element Carbon (C), which exhibits two isotopes, one with a mass of 12 and an abundance of 98.9%, and the other with a mass of 13 and an abundance of 1.1%. The μ domain function for C is delineated in Supplementary equation 9. By extending this methodology, proceed to deduce the μ domain function, denoted as $f_i(\mu)$, for the diverse elements comprising the molecule.

$$f_c(\mu) = 0.989e^{2\pi i*12\mu} + 0.011e^{2\pi i*13\mu} \tag{9}$$

The μ domain function $f(\mu)$ for the molecular formula can be derived by considering the μ domain functions of each constituent element and the quantity of each element present. Taking CO_2 as an example, there is one carbon atom and two oxygen atoms in this molecule. Based on this information, the μ domain function for the molecular formula CO_2 can be computed, as demonstrated in Supplementary Equation 10.

$$f_{co_2}(\mu) = f_c(\mu)f_o^2(\mu) \tag{10}$$

Conducting an Inverse Fast Fourier Transform (IFFT) on the peak shape function $S(n)$ in the quality domain yields the peak shape function $s(\mu)$ in the μ domain. Throughout this process, we can extract all necessary data by executing a unilateral Fourier transform on the Gaussian function, leveraging its symmetry and real-number properties. Based on the characteristics of Fourier transforms, the convolution in the mass domain is analogous to multiplication in the μ domain. Consequently, the ultimate μ domain function is presented in Supplementary Equation 11.

$$\varphi(\mu) = s(\mu)f(\mu) \tag{11}$$

Finally, concerning $\varphi(\mu)$, after sampling, perform a Fast Fourier Transform (FFT) and convert it to the quality domain, $\psi(m)$. This process allows for the determination of the theoretical isotope distribution of the molecular formula in the mass domain.

Supplementary Note 2：The Impact of SMILES and SELFIES Representation on Model Predictive Performance

The SMILES representation is one of the most commonly used ways to express a chemical molecular structure. However, its drawback lies in the fact that some strings may not correspond to valid molecules. To explore the impact of this drawback on the model, we selected SELFIES (Self-Referencing Embedded Strings), a string-based molecular representation with 100% robustness, where each SELFIES string corresponds to a valid molecule. We trained the Transformer using two different molecular representations, and the cross-entropy loss during training is depicted in Supplementary Figure S1, showing both methods converge well with no significant differences. The detailed data is available in Supplementary Data 3 of the "Supplementary Data.xlsx". Subsequently, we compared the fingerprint similarity distribution of predicted molecules in the test set, as shown in Supplementary Table S2. It is observed that SELFIES representation is slightly inferior to SMILES in the most similar results (Similarity=1) but exhibits some improvement in other cases.

Despite the instability of SMILES representation, which may generate invalid SMILES strings in a single prediction, this uncertainty is mitigated through the model's repeated predictions. Additionally, it is important to note that the primary focus of this study is to combine deep learning models with subsequent fragment tree models for accurate inference of target substances. The choice of which representation to apply does not significantly impact the experimental results. In conclusion, this study adopts SMILES strings for molecular characterization as it aligns well with our overall objectives. Future research can explore the merits and drawbacks of alternative representation methods.

Supplementary Figure S1: The cross-entropy loss reduction curves for two representation methods during training

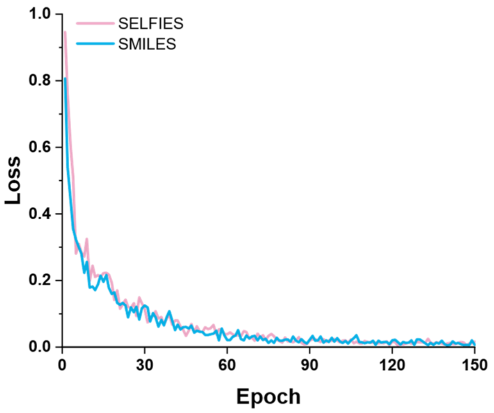

Supplementary Table S2: Fingerprint similarity distribution of prediction results using different molecular representation methods

Tanimoto similarity	SMILES	SELFIES
Similarity=1	29.5%	27.1%
0.9⩽Similarity<1	17.6%	31.4%
0.8⩽Similarity<0.9	19.4%	22.1%

Supplementary Note 3：The influence of different Transformer parameters on model performance

Considering the on-site detection scenarios and server performance constraints, we sought a relatively lightweight model architecture for substance prediction. To find the most suitable parameters, we experimented with different numbers of decoder and encoder layers in the Transformer model, as well as varying mass resolution of the mass spectrum input. The performance comparison is shown in Supplementary Table S3. Ultimately, we selected the Base-Transformer model. Additionally, we compared the impact of different optimizers (Adam and SGD) on the predictive performance of the Transformer model, as presented in Supplementary Table S4. It is observed that the choice of optimizer has a minimal impact on the model's performance.

In terms of the encoding format for mass spectrum data, we explored the impact of different numerical encoding (NE) methods on the model. The first encoding format, utilized in this study, involves constructing a one-hot matrix using truncated m/z peaks. For each m/z peak, we truncated it based on a resolution of 0.01 Da. This encoding method effectively preserves the ordering and relationships between numerical values. During the dataset creation process, substances with a relative molecular mass exceeding 500 were excluded to reduce the dimensionality of the one-hot matrix. Despite the relatively large final dimension of this one-hot matrix, it is deemed necessary for the model training process. Additionally, we conducted ablation experiments by altering the numerical encoding method, for instance, using Vaswani's definition (as shown in Supplementary equations 12 and 13) as the numerical encoding to train the Transformer model. d_e is the embedding size.

$$NE(v,p,2j) = \sin\left[(v * 10^p)/10000^{2j/d_e}\right] \quad (12)$$

$$NE(v,p,2j+1) = cos\left[(v * 10^p)/10000^{2j/d_e}\right] \quad (13)$$

Unfortunately, adopting this encoding method did not improve the convergence of the model; on the contrary, it tended to result in discreteness. The changing trend of cross-entropy loss values, as illustrated in Supplementary Figure S2, indicates a significant negative impact of this numerical encoding method on the model. Therefore, we adhere to the first numerical encodings.

Supplementary Table S3: The selection of Transformer parameters

	Mass resolution	Number of Encoder and Decoder Layers	Time	RAM	Cross-Entropy Loss	Accuracy
Large-Transformer	0.01Da	12	113.3h	10GB	$3.61*10^{-3}$	Similarity >0.5 90.83%
						Similarity >0.8 69.10%
Base-Transformer	0.01Da	6	21.4h	7GB	$1.61*10^{-3}$	Similarity >0.5 96.00%
						Similarity >0.8 75.60%
Small-Transformer	0.1Da	6	20h	2GB	$1.69*10^{-2}$	Similarity >0.5 86.40%
						Similarity >0.8 24.40%

Supplementary Table S4: The selection of optimizers.

Base-Transformer	Time	Accuracy	
SGD	20.9h	similar>0.5	97.40%
		similar>0.8	74.50%
Adam	21.4h	similar>0.5	96.00%
		similar>0.8	75.60%

Supplementary Figure S2: The cross-entropy loss reduction curves for two numerical encoding methods during training

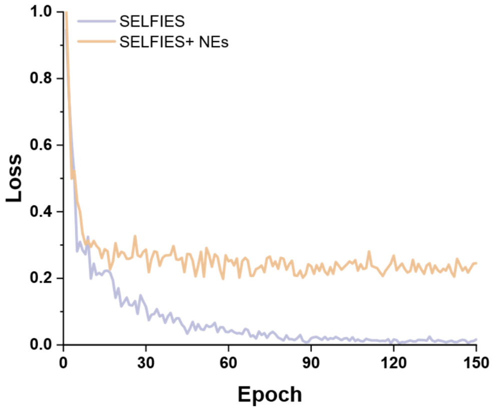

Supplementary Note 4：The influence of different decoding strategies

In terms of the model's decoding strategy, beam search is the most commonly used method, while another decoding strategy is greedy decoding. Greedy decoding can be considered a special case of the beam search decoding method with a beam width of 1. As the beam width increases, the model can rapidly generate a greater variety of compounds in a single prediction. To investigate the impact of beam width on the model's decoding, we compared the prediction results of greedy decoding and beam search with a beam width of 3. Experimental data indicate that the beam width has no significant effect on the final experimental results. The model's outputs consistently tend to resemble several molecules closely related to those generated by the greedy decoding method. Furthermore, analyzing the similarity of the final output results reveals that, although the beam search decoding method can generate a greater variety of molecules, the similarity does not show a significant improvement. In conclusion, this experiment adopts greedy decoding as the model's decoding strategy.

Supplementary Table S5: Fingerprint similarity distribution of prediction results using different decoding strategies

Tanimoto similarity	Greedy decoding	Beam search (Beam width=3)
Similarity=1	27.2%	27.2%
0.9⩽Similarity<1	31.4%	29.8%
0.8⩽Similarity<0.9	22.1%	24.4%

Supplementary Note 5: The detail of molecular re-prediction

Taking Pterostilbene as an example, we elaborate on how to improve the molecular similarity through repeated predictions. Firstly, using the TeFT model, we predict and score the MS^2 spectrum of Pterostilbene. In the scoring results, we find that the structure 1 in the highest-scoring tree (as shown in Supplementary Figure S3 left) also appears in the trees with higher rankings. Simultaneously, the molecular weight of this structure is close to a peak in the fragmentation spectrum of Pterostilbene. Considering the fragmentation properties of stilbene compounds, we hypothesize that this structure could be a substructure of the target compound. Since the SMILES representation of a molecule allows arbitrary designation of the starting atom, we can expand upon this fragment based on this characteristic. In the second prediction process, the SMILES string of this fragment and the MS^2 spectrum of Pterostilbene are both input into the model. The Transformer model continues generating molecules based on the input string, allowing the model to further expand upon this fragment. Ultimately, the model successfully predicts the complete structure of Pterostilbene based on this fragment.

Supplementary Figure S3: The re prediction process of Pterostilbene

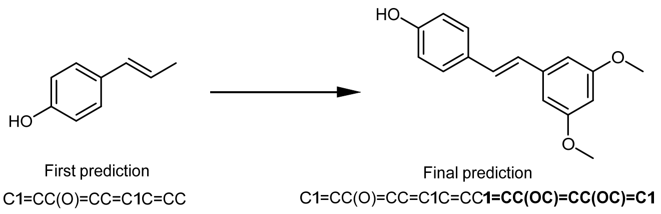

Supplementary Figure S4: Top-5 accuracy results for two benchmark methods and TeFT. The accuracy of the top-k methods for the three approaches in the test set, where k varies from 1 to 5. The detailed data is available in Supplementary Data 4 of the "Supplementary Data.xlsx".

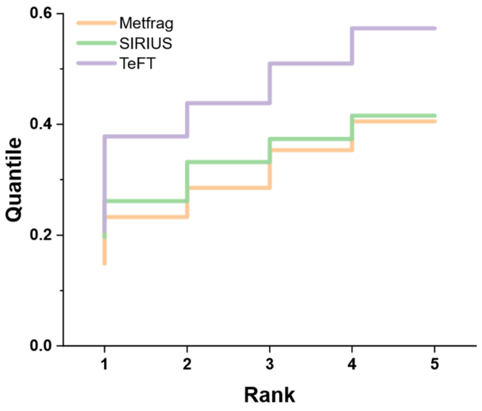

Supplementary Figure S5: MS^2 spectra of other peaks of Anweiyang capsules. Average MS^2 spectrum of m/z=262 and 249 peak using high-resolution mass spectrometer.

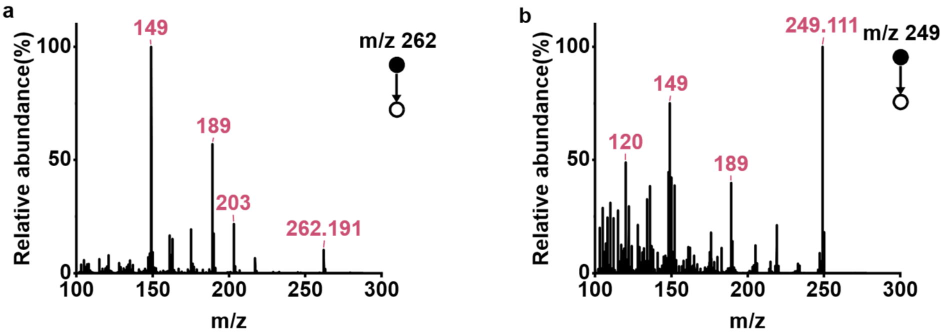

Supplementary Table S6: Evaluate model performance using the top-k method. Using the SIRIUS4 and MetFrag methods as replacements for the Transformer component of TeFT, predict potential SMILES results for the mass spectrum. Subsequently, generate fragment tree models, compare similarities, and calculate the proportion of substances with the highest similarity, ranking first.

Model	Top-1 accuracy
TeFT	35.8%
SIRIUS4 + Tree score	27.9%
MetFrag + Tree score	26.7%
SIRIUS4	29.9%
MetFrag	24.9%
