# Supplementary material for: Structural annotation of unknown molecules in a miniaturized mass spectrometer based on a transformer enabled fragment tree method
Source: Commun Chem. 2024 May 13;7:109. doi: 10.1038/s42004-024-01189-0 (PMC11091078; doi:10.1038/s42004-024-01189-0)
Supplement: Supplementary file 3 — Description of Additional Supplementary Files [file 42004_2024_1189_MOESM3_ESM.pdf]

Description of Additional Supplementary Files

File name: Supplementary Data 1

Description: The Tanimoto similarity of the best incorrect candidate to correct structure for TeFT, SIRIUS4, and MetFrag in different datasets.

File name: Supplementary Data 2

Description: Model performance in Miniaturized Linear Ion Trap Mass Spectrometer. Test results for all substances.

File name: Supplementary Data 3

Description: The cross-entropy loss reduction curves for two numerical encoding methods and two representation methods during training

File name: Supplementary Data 4

Description: The accuracy of the top-k methods for the three approaches in the test set, where k varies from 1 to 5
